# The Other T Helper Cells in Asthma Pathogenesis

**DOI:** 10.1155/2010/519298

**Published:** 2010-07-25

**Authors:** Christina Vock, Hans-Peter Hauber, Michael Wegmann

**Affiliations:** ^1^Department of Experimental Pneumology, Research Center Borstel, 23845 Borstel, Germany; ^2^Medical Clinic, Research Center Borstel, 23845 Borstel, Germany

## Abstract

The complex phenotype of allergic bronchial asthma involves a variable degree of bronchoobstruction, increased mucus production, and airway remodeling. So far it is suggested that it arises from multiple interactions of infiltrating and structural cells in the context of chronic airway inflammation that is orchestrated by T helper 2 (TH2) cells. By secreting a plethora of typical mediators such as interleukin (IL) 4, IL-5, and IL-13, these cells hold a key position in asthma pathogenesis. However, therapeutic approaches targeting these TH2-type mediators failed to improve asthma symptoms and impressively showed that asthma pathogenesis cannot be reduced by TH2 cell functions. Recently, other T helper cells, that is, TH9 and TH17 cells, have been identified and these cells also contribute to asthma pathogenesis, the processes leading to formation or aggravation of asthma. Furthermore, TH25 cells, TH3 cells, and regulatory T cells have also been implicated in asthma pathogenesis. This paper aims at summarizing recent insights about these new T helper cells in asthma pathogenesis.

## 1. Introduction

A variable degree of bronchoobstruction based on airway hyperresponsiveness (AHR) and allergen-dependent mast cell degranulation together with chronic airway eosinophilia increased mucus production, and airway remodeling sketches the typical pathologic picture of allergic bronchial asthma [1]. Currently, this complex phenotype is believed to arise from manifold interactions of infiltrating immune cells with structural cells of the airways. Although these processes comprise a plethora of cells such as T cells, B cells, mast cells, and macrophages on the one hand and smooth muscle cells, fibroblasts, and airway epithelial cells on the other hand, over the last 15 years a subpopulation of CD4+ T cells emerged as key players in asthma pathogenesis—the T helper 2 (TH2) cell. By releasing a number of typical cytokines, these cells orchestrate a number of inflammatory events that subsequently trigger cascades of other processes ultimately leading to the formation of the disease. These typical cytokines involve among others especially interleukin 4 (IL-4), IL-5, and IL-13 which have all been detected in increased amounts in asthmatic patients [2].

IL-4 upregulates the fate-determining transcription factor GATA-3 in naïve T helper cells and is therefore essential for the initial differentiation and expansion of allergen-specific TH2 cells [3]. Besides its importance for TH2 cell development, IL-4 further plays a significant role in establishing the basis for IgE-mediated allergic reactions. Thus, IL-4 is one of two essential factors inducing the isotype class switch in plasma cells from the production of IgM to IgE; the other factor is IL-13 [4, 5]. Furthermore, IL-4 triggers the expression of both the high and the low affinity Fc*ε* receptors [6]. Besides its affects on differentiating B cells IL-5 predominantly works on eosinophils by effecting their differentiation, recruitment, activation, and survival in the periphery predisposing this cytokine as a central factor in regulating airway eosinophilia in asthma [7]. In contrast to IL-4 and IL-5 that mainly act on immune cells, IL-13 impacts airway epithelial cells and smooth muscle cells, where it mediates mucus hypersecretion, subepithelial fibrosis, and development of AHR [8]. By secreting these three cytokines, TH2 cells exert influence on asthma pathogenesis on the level of T cells, B cells, and structural cells. The importance of these factors has further been figured out in mouse models of experimental asthma: neither in animals deficient for IL-4 or IL-13 nor in animals deficient for the IL-5 receptor it was possible to induce allergic airway inflammation or AHR [9–11]. In line with these observations, neutralizing these mediators with specific antibodies not only prevented the development of experimental asthma but also diminished its phenotype in already diseased animals [12].

Despite these promising results, the clinical approaches towards asthma therapy by neutralizing IL-4, IL-5, or IL-13 frustrated the high expectations or did not progress to clinical trials [13–15]. These studies strikingly demonstrated the complexity of the pathogenetic mechanisms underlying the formation of allergic bronchial asthma and lead to a discussion about the proposed predominant role of TH2 cells within these processes. Additionally, over the last few years further T helper cell subsets are identified, namely, TH9 and TH17 cells, which also seem to be involved in asthma pathogenesis. Although TH1 cells have been shown to act as opponents to TH2 cells that is even able to counteract ongoing allergic immune responses [16], there exist a few studies that clearly demonstrated a participation of interferon *γ* (IFN-*γ*) in the development of AHR. This paper aims at summarizing the latest developments of T helper cell research in asthma research.

## 2. TH9 Cells in Asthma Pathogenesis

TH2 cells have been regarded as a major source of IL-9 production [17]. Several studies showed IL-9 mRNA and protein expression in the lymphocytes of bronchoalveolar lavage and CD3+ T cells in bronchial tissue from asthmatics [18, 19]. However, recent studies described a new subset of CD4+ T cells distinct from TH2 cells. These cells were named TH9 cells because they produce IL-9 in large amounts [20, 21]. 

Different cytokines can influence the differentiation of naïve CD4+ T cells into different subtypes. Transforming growth factor *β* (TGF-*β*) can “reprogram” the differentiation of T helper cells to an IL-9 producing phenotype, the so-called TH9 cell [20]. In a study by Veldhoen and colleagues, TGF-*β*1 together with IL-4 was able to drive highly polarized naïve T cells (CD4+CD25-CD44^low^) into TH9 cells in the absence of IL-6. TGF-*β*1 caused a loss of the TH2 fate-determining transcription factor GATA-3 and consequently loss of expression of TH2 type cytokines IL-4, IL-5, and IL-13 in those TH2 cells. Since loss of GATA-3 by conditional gene deletion abolishes the production of IL-5 and IL-13 but not IL-4, it is unlikely that the effect of TGF-*β* simply mimics the loss of GATA-3 [22]. These TH9 cells produce IL-9 and IL-10. Although IL-10 may promote IL-9 production in human and mouse T cells [23, 24], blockade of the IL-10 receptor did not compromise the ability of these cells to produce IL-9. 

Another study found that IL-4 could inhibit the generation of TGF-*β*-induced Treg cells expressing the transcription factor Foxp3^+^. That study by Dardalhon and coworkers used naïve CD4+Foxp32-CD62L+ T cells from mice. Surprisingly, the combination of IL-4 and TGF-*β* generated Foxp3^−^ effector T cells producing IL-9 and IL-10 [21]. In contrast to the role of IL-10 as an anti-inflammatory cytokine, these cells induced colitis and neuritis in recombination-activating gene- (RAG-) 1-deficient mice [21]. A more recent study by Jäger and colleagues showed that TH9 cells could induce experimental autoimmune encephalomyelitis upon adoptive transfer in mice [24]. So far, no defined transcription factor has been found in TH9 cells as compared to TH2 T cells (GATA-3), TH1 cells (T-bet), or TH17 cells (ROR*γ*t). It has also to be taken into account that the above-mentioned studies used mouse models. Whether TH9 cells are present in humans or not is not proven yet. 

It is interesting that IL-9^+^IL-10^+^Foxp3^−^ T cells do not possess anti-inflammatory activities or immunoregulatory properties despite their production of IL-10 [25–27]. These TH9 cells have to be considered as effector T cells. However, their role in health and disease is not clear at the moment, even if experimental approaches have shown that they can induce autoimmune encephalomyelitis [24]. To understand the possible role of IL-9 producing cells, it has to be asked what role IL-9 plays in physiologic and pathologic conditions.

IL-9 belongs to the family of 4-helix bundle cytokines. This family also includes IL-2, IL-3, IL-4, IL-6, IL-7, and IL-15. Human IL-9 consists of a 14-kd glycoprotein, the mature form of which is composed of 144 amino acids along with a signal sequence of 18 amino acids. The IL-9 protein contains a high proportion of cationic amino acid residues and 10 cysteines and has 4 N-linked glycosylation sites. The IL-9 gene is located on chromosome 5 (5q31-35) next to the genes encoding IL-3, IL-4, IL-5, IL-13, CD14, and granulocyte-macrophage colony-stimulating factor [28]. All of these mediators have been implicated in allergic inflammation [29].

Besides T cells IL-9 production has been reported in several other cell types including mast cells, eosinophils, and neutrophils [18, 30–34]. In asthmatic patients, mast cells, eosinophils, and neutrophils have been found to express IL-9 besides T cells. 

IL-9 was originally described as a potent T cell and mast cell growth factor in mice [35]. It has pleiotropic effects on various cell types. IL-9 enhances the survival of mast cells and induces production of IL-6 [30, 35]. In addition, IL-9 stimulates the production of mast cell proteases and the expression of the high-affinity IgE receptor [Fc*ε*RI*α*] [36], suggesting that IL-9 may prime mast cells to respond to allergen challenge through increased expression of Fc*ε*RI*α* and increased production of IL-6 and proteases. In T cells, IL-9 acts as a growth factor by supporting antigen-independent growth of T helper cell clones [37]. Moreover, IL-9 inhibits lymphokine production by IFN-*γ* producing CD4+ T cells and promotes proliferation of CD8+ T cells [38]. Transgenic IL-9 overexpressing mice have increased levels of immunoglobulin isotypes including IgE and expansion of lymphocytes from the B1 subset (Mac^+^IgM^+^). Furthermore, accumulation of B cells in the lungs and airway infiltration with T cells could be observed in the animals [39–41]. IL-9 promotes eosinophil maturation in synergy with IL-5 [42]. Soussi Gounni and coworkers showed that IL-9 increased the expression of the IL-5 receptor *α*-chain (IL-5R*α*) on eosinophils [43]. In that study, IL-9 inhibited eosinophil apoptosis and enhanced eosinophil development. Expression of the IL-9 receptor *α*-chain (IL-9R*α*) has also been demonstrated on neutrophils and IL-9 induced the production and release of IL-8 by neutrophils from asthmatic subjects [43]. Epithelial cells release several chemokines and cytokines upon stimulation with IL-9 [44, 45]. Furthermore, IL-9 has been shown to stimulate mucin transcription and to upregulate mucus expression in airway epithelial cells [46, 47].

As mentioned above, the IL-9 encoding gene has been localized in the chromosomal region 5q31-33 which has been identified to contain several candidate genes for airway hyperresponsiveness (AHR) [28, 29]. Nicolaides and colleagues were the first to show a close association between the IL-9 gene and bronchial hyperresponsiveness [48]. In that study, analysis of the murine IL-9 gene identified a genetic defect at the C57BL/6(B6)IL-9 locus associated with a lack of IL-9 expression in the lung and reduced AHR in naive B6 mice. In that same study, an increased expression and production of IL-9 in the lung was associated with AHR in DBA/J2 mice. The tight genotype-phenotype relation was further supported by the finding that (B6D2)F1 mice were found to be intermediate in both lung IL-9 levels and airway responsiveness. Selective overexpression of the IL-9 gene in the airways of transgenic mice resulted in massive airway inflammation, with infiltration of eosinophils and lymphocytes, mast cell hyperplasia, and increased subepithelial collagen deposition [39, 44, 49]. Moreover, elevated IgE levels, AHR, and increased responsiveness to antigen stimulation could be observed. In contrast, overexpression of IL-4 using the CC-10 promoter resulted in baseline eosinophilia without AHR [50]. Instillation of recombinant mouse IL-9 into the airways of B6 mice for up to 10 days resulted in time-dependent and dose-dependent increases in AHR, BAL eosinophilia, elevated IgE levels, increased lung proteases, and submucosal membrane thickening [51]. In a study by Shimbara and colleagues, both the degree of airflow obstruction (FEV1) and airway responsiveness to metacholine significantly correlated with IL-9 mRNA expression [18].

Airway remodelling is a hallmark of chronic bronchial asthma. It is characterized by structural changes of the airways including epithelial mucus metaplasia, peribronchial fibrosis, increases in airway smooth muscle mass, and angiogenesis [52]. These changes are mediated by chemokines and cytokines [53]. IL-9 has been shown to induce mucus expression. Both in vitro and in vivo studies have demonstrated that IL-9 can directly induce metaplasia independent of inflammation [54, 55]. However, some studies have suggested that IL-9-induced mucus expression is mediated via IL-13 [56, 57]. In IL-9 transgenic mice, subepithelial fibrosis was observed, but this may be due to mediator release and cell recruitment [58]. IL-9 appears to play an important role in airway remodelling especially with epithelial mucus production. Doherty and colleagues have shown that CD4+ T cells, are not required for remodelling after establishment of acute inflammation [59]. CD4+ T cells remain essential for eosinophilic inflammation even during late antigen challenges. It is tempting to speculate that IL-9 secreted from CD4+ T cells may play an important role in early initiation of airway remodelling. However, other cells and cytokines are likely to contribute later. Airway remodelling in human asthmatics and in mouse models may be regulated by TGF-*β* on many levels [60]. Together with IL-4 this may provide the adequate “milieu” to differentiate or possibly “reprogram” effector T cells into TH9 cells. 

Although animal models have shown that TH9 cells may represent a new subtype of T cells information on their role in human disease is speculative at the moment. So far they have not been described in humans. One has to keep in mind that several inflammatory cells can express IL-9 in bronchial asthma. Therefore, the importance of these “new” cells has to be regarded with caution. The first step will be the identification of TH9 cells in human patients. Nevertheless, the role of IL-9 as an important TH2 type cytokine in bronchial asthma and other diseases (e.g., allergic, autoimmune) cannot be questioned. 

## 3. TH17, TH17/TH2, and TH22 Cells in Asthma Pathogenesis

A few years ago, the identification of TH17 cells as a new distinct subtype of T helper cells nearly overthrew the established TH1/TH2 paradigm, so that these cells developed to a hot spot of actual immunological research. Characterized by the production of the eponymous cytokine IL-17A and the expression of retinoic acid-related orphan receptor *γ*t (ROR*γ*t) (in mice) or ROR c in humans, respectively, these cells were observed for the first time in a mouse model of autoimmune diseases like experimental autoimmune encephalitis or collagen-induced arthritis [61, 62]. Subsequent studies identified these cells as potent producers of well-known proinflammatory mediators such as tumor necrosis factor *α* (TNF-*α*), IL-1*β*, and IL-6 and of relatively new cytokines like IL-17A, IL-17F, IL, 21, IL-22, and IL-26, pushing these cells into the role of general promoter of inflammatory processes [63–65].

In retrospect first hints towards participation of TH17 cells in asthma pathogenesis were reported in 2001 in two studies that detected increased amounts of IL-17 (that is named IL-17A today) in plasma samples [66] and an increased expression of IL-17 mRNA in airway tissues of asthmatic patients [67]. However, identification of TH17 cells in asthmatic airways succeeded not until 2008. In a study by Péne et al., the author, detected highly activated TH17 cells in bronchial biopsies of patients suffering from severe asthma, where these cells accounted for not less than 20% of all infiltrating lymphocytes. In vitro restimulation of these cells resulted in production of IL-17A and IL-17F, IL-22, IL-26, lymphotoxin *β* (LT-*β*), and TNF-*α* [68]. Together with the finding that increased levels of IL-17A mRNA correlate with increased numbers of neutrophils in sputum samples of asthmatic patients, these results suggested TH17 cells to play a key role in asthma aggravation towards a severe phenotype by recruiting neutrophils to already inflamed asthmatic airways [69]. This hypothesis is supported by the finding, that human TH17 cells induce the in vitro production of IL-8, together with IL-6, TNF-*α*, granulocyte-macrophage colony-stimulating factor (GM-CSF), and growth-related antigen *α* (GRO-*α*) in human bronchial epithelial cells [70–72] and human bronchial smooth muscle cells [73–75] by secreting IL-17A and IL-17F. TH17 cells are further capable of activating neutrophils by secreting IL-6, TNF-*α*, IL-1*β*, and IL-17A, which act synergistically on the production and activity of human neutrophil elastase (HNE) and myeloperoxidase, two of the most prominent neutrophil-derived enzymes [76]. At least, TH17 prolongs the survival of neutrophils by releasing GM-CSF and granulocyte colony-stimulating factor (G-CSF), enabling them not only to promote but also to sustain airway neutrophilia [77].

Although TH17 cell development in humans and mice differs in some points, the link between TH17 cells and neutrophil infiltration into the lung could be confirmed by several mouse studies. First indications were observed in two studies that aimed at eliminating CD4+ T cells from mice with experimental asthma by using monoclonal antibodies, against CD4 or IL-2, respectively. In both studies ablation of T helper cells did not inhibit notonly airway eosinophilia but also neutrophil infiltration [78, 79]. Moreover, intranasal application of IL-17A resulted in pronounced neutrophil accumulation and increased levels of active matrix metallo protease 9 (MMP-9), another typical neutrophil-derived enzyme, in broncho-alveolar lavage (BAL) [80]. Consequently, adoptive transfer of in vitro generated OVA-specific TH17 cells into transgenic DO11.10 mice with acute experimental asthma induced marked airway neutrophilia and unresponsiveness to treatment with cortico steroids [81]. Two other studies strongly suggest that such effects of TH17 cells seem to be mediated through release of IL-17A and IL-17F [82, 83]. These experimental results strongly suggest a role for TH17 cells in the development of airway neutrophilia in severe asthma. Clinical observations additionally support this hypothesis. Thus, IL-17A is expressed in the airways of asthmatic patients and correlates with the numbers of infiltrating neutrophils and disease severity [67, 84]. The question whether these cells play a role in mild-moderate asthmatics as well remains to be answered, especially since two recently published studies demonstrated the appearance of IL-17A and IL-22 producing TH17 cells in peripheral blood of asthmatic patients [85, 86]. Most recently, another T helper cells population has been described that produce not only IL-17A, IL-21, and IL-22 but also considerable amounts of the typical TH2-type cytokines IL-4, IL-5, and IL-13. Consequently, these cells were named TH17/TH2 cells (Cosmi, 2010). Because of these special functional properties and the increased numbers in the circulation of asthmatic patients, this new T helper cell subset also awaits characterization of its role in asthma pathogensis.

Furthermore, it remains also unanswered whether TH17 cells are the only source of IL-17s within the processes leading to airway neutrophilia in asthmatic lungs since CD11b+/F4/80+ alveolar macrophages are also capable of producing considerable amounts of IL-17A as demonstrated in a mouse model of experimental asthma [87]. Furthermore, distinct subtypes of *γ* *δ* T cells, CD8+ T cells, and natural killer (NK) cells have also been shown to secrete IL-17A even in larger amounts as TH17 cells [88–91]. By doing so, at least NK cells appear to be involved in the development of airway neutrophilia and AHR in a mouse model of ozone-induced experimental asthma [92].

However, these studies investigated just one aspect of TH17 cell activity while other functions and effects during asthma pathogenesis, progression, or aggravation remain unclear. As already mentioned, TH17 cells isolated from bronchial biopsies of severe asthmatics appeared to be highly activated and secreted a plethora of proinflammatory cytokines after in vitro restimulation. Although cytokines with strong proinflammatory potential such as TNF-*α* and IL-6 have meanwhile been implicated in at least several aspects of asthma pathogenesis [93–95], the effects of other TH17 cell derived factors such as IL-17F, IL-22, and IL-26 have only been investigated fragmentarily. 

IL-17F belongs to the IL-17 family and is approximately 50% homologous with IL-17A, more than with any other member of this family. Moreover, the genes encoding IL-17A and IL-17F lie next to each other on chromosome 6 [96]. Its proinflammatory potential has been characterized in in vitro airway epithelial cells and fibroblasts where it induced the expression of various cytokines, chemokines, and adhesion molecules such as IL-6, IL-8, GRO*α*, epithelial cell-derived neutrophil-activating protein 78 (ENA-78), transforming growth factor *β* (TGF-*β*), G-CSF, GM-CSF, monocyte chemotactic protein, and intracellular adhesion modelcule 1 (ICAM-1) [74, 97, 98]. These observations do not only suggest that both genes arise from a gene duplication event but also that both gene products could have similar functions. Increased expression of IL-17F has already been detected in asthmatic patients after allergen challenge. Interestingly, the authors did not observe any augmented expression of IL-17A in the same study [99]. Pulmonary overexpression of IL-17F in mice resulted not only in airway neutrophilia but also in an amplified allergen-induced immune-response including increased airway eosinophilia, AHR, and goblet cell hyperplasia [83]. The question how IL-17F affects the allergic immune-response in the lung has so far not been answered. However, lung tissue of these transduced animals revealed markedly increased levels of several proinflammatory cytokines and chemokines such as IL-6, IFN-*γ*, inflammatory protein 10, monokine, insulin-like growth factor, and C10, demonstrating the proinflammatory potential of IL-17F in vivo.

IL-21 belongs to the IL-2 cytokine superfamily and is produced by several T cell subpopulations such as TH2 cells, follicular B helper T cells, and TH17 cells, while the last appears to produce the largest amounts [100–103]. The expression of this cytokine by TH17 cells depends on the signal transducer of activated T cells 3 (STAT-3) and is strongly induced by IL-6 [104]. Together with IL-6 or TGF-*β*, IL-21 is able to promote TH17 cell differentiation by inducing the expression of ROR*γ*t. Therefore, this cytokine is suggested to play a critical role in amplifying a TH17 cell response as it has been demonstrated for IFN-*γ* for TH1 cells or IL-4 for TH2 cells respectively [105]. Another activity of IL-21 that might play a role in asthma pathogenesis involves regulation of IgE-production in plasma cells [106].

IL-22 and IL-26 are members of the IL-10 cytokine superfamily. So far, IL-22 expression has been detected in activated T helper cells and, to a lesser degree, inactivated NK cells or so-called NK22 cells, whereas it is absent in dendritic cells, macrophages, and nonimmune cells [107]. Interestingly, the IL-22 receptor has yet not been detected in cells of the immune system, but in a broad range of nonimmune cells including epithelial and endothelial cells. This observation leads to the hypothesis that IL-22 serves for the communication between TH17 cells and tissues in order to form up the immune barrier function of epithelia against bacterial infections and to induce production of antibacterial peptides [108–110]. Recently, a small subpopulation of skin-homing CD4+ T cells has been identified produced IL-22 as well but lack the production of IL-17 or IFN-*γ* [111]. Furthermore, these cells did not express the TH17-typical transcription factor ROR*γ*t suggesting these cells as a T helper cells subpopulation that is distinct from TH17 cells thus representing a new group called TH22 cells. It remains to be seen whether those TH22 cells will be identified in asthmatic patients as well. IL-26 is clustered together with IL-22 on chromosome 12q15 and is primarily produced by T cells and monocytes. Up to now it has been implicated in virus-induced transformation of T cells [112]. However, the contribution neither of IL-22 nor of IL-26 to asthma pathogenesis has been understood so far.

The question for TH17 recruitment to the lung or their differentiation in asthmatic airways remains unanswered as well. Since IL-23 is required for the full acquisition of the pathogenic function of human and murine TH17 cells the “IL-23-TH17-axis” which has been established in severe atopic dermatitis has also been hypothesized for asthma. This hypothesis was supported by an in vivo study showing that airway infiltrating TH17 cells together with local IL-23 expression significantly enhanced eosinophilic airway inflammation in a mouse model of experimental asthma [113]. An in vitro study performed by Cheung et al. further suggested a role for IL-23 in asthma pathogenesis. The authors demonstrated that IL-23 in combination with IL-17A and IL-17F promoted the production of CXCL1 and IL-8 in human eosinophils generating a strong neutrophilotactic signal, whereas IL-23 together with IL-17F alone induced the production of IL-1*β* and IL-6 which could reciprocally continue TH17 cell activation [114]. These data strongly suggest a close relationship between IL-23 on the one hand and TH17 cell development and/or activation on the other hand in the pathogenesis of severe asthma.

Whether TH17 cells could also differentiate in a TH2 cell dominated surrounding was answered by a study carried out in vitro by Tanaka et al. By using CD11+ dendritic cells stimulated with human thymic stromal lymphopoetin (TSLP) and the artificial TLR-3 ligand poly IC the others were able to induce TH17 cells with a central memory phenotype even under TH2-polarizing conditions [115]. Since TLR-3 originally recognizes double stranded RNA which appears as an intermediate during viral replication, this study supports the hypothesis that recurrent viral infections, which are clinically correlated with asthma aggravation towards a severe phenotype, play a role in TH17 cell recruitment to asthmatic airways.

## 4. TH25 Cells in Asthma Pathogenesis

IL-25 belongs to the IL-17 cytokine family and is also called IL-17E but induces a cytokine profile that is completely different from those triggered by IL-17A or IL-17F. Its expression has been reported in activated TH2 cells (Fort, 120)—recently suggested as a new subpopulation of T helper cells, the TH25 cells [116]. Transgenic overexpression in mice resulted in airway eosinophilia and elevated serum levels of the typical TH2-type cytokines IL-4, IL-5, and IL-13 together with increased IgE serum titers [117, 118]. Similar results were obtained by intranasal administration of an IL-25-expressing adenovirus vector. When IL-25 was administered intraperitoneally, additionally development of AHR, increased mucus production, and epithelial cell hyperplasia could be observed [119]. Conversely, mice deficient in IL-25 exhibit reduced TH2-type immune responses [120, 121] and blocking IL-25 by the use of a monoclonal antibody or by a soluble IL-25 receptor (sIL-25R) prevented from development of AHR, production of allergen-specific IgE, allergic airway inflammation, and increased mucus production in a mouse model of experimental asthma [122, 123].

Studies revealed that IL-25 predominantly affects a non-T/non-B cells population expressing class II MHC and CD11c molecules and drives production of IL-4, IL-5, and IL-13, which consequently induces TH2-type immune responses. While these cells exhibit the characteristics of a typical accessory cell type, most recently a subset of invariant natural killer T cells (iNKT cells) expressing IL-17RB, a receptor for IL-25, have also been identified as a target for IL-25. These cells produced large amounts of TH2-type cytokines after stimulation with IL-25 and were capable of restoring AHR in mice deficient for iNKT cells [124]. Another study demonstrated that in vitro IL-25 induced the production of IL-9 that is independent from IL-4 in IL-17R expressing T cells [125].

While these studies clearly demonstrated that IL-25 is involved in the initiation of allergic diseases, the study of Tamachi et al. showed that IL-25 enhances allergen-induced airway inflammation by amplifying the already established TH2-type immune-response [123]. With its amplifying impact on TH2 cells IL-25 shares in part functional similarities with another novel cytokine, termed IL-33. This is produced by many tissue related cell types including epithelial cells, endothelial cells, smooth muscle cells, keratinocytes, fibroblasts, and adipocytes. It broadly enhances allergic inflammation through its effects on hematopoietic cell types that is, it triggers the production of the typical TH2-type cytokines IL-4, IL-5, and IL-13 in T cells, NKT cells, basophils, and mast cells or prolongs the survival of eosinophils [126, 127].

## 5. Regulatory T cells and TH3 Cells in Asthma Pathology

Regulatory T cells (Tregs) and TH3 cells have both been described as CD4+ T cells that also express the alpha chain of the IL-2 receptor (CD25) and that act to suppress immune responses by releasing anti-inflammatory cytokines such as TGF-*β* and IL-10. While Tregs require IL-2 for their survival, in vitro differentiation of TH3 cells depends on TGF-*β* so that these cells have been suggested to form another T cell subpopulation [128]. Regulatory T cells have been suggested to be critical important for maintaining self-tolerance in order to prevent autoimmunity, they further suppress allergy, asthma, and pathogen-induced immune-pathology, and they seem to regulate oral tolerance as well as feto-maternal tolerance. The role of these cells in the immune-pathogenesis of allergic asthma has been extensively reviewed elsewhere and will only be discussed here in brief [129, 130].

The basis for atopic sensitization and the subsequent development of an allergic disease such as bronchial asthma is an inappropriate TH2 cell response to common antigens. The answers that try to address the question why asthmatic patients develop typical allergic airway inflammation range from genetic susceptibility and coexposure to infectious agents to variations in the dose and timing of allergen exposure. However, over the last 10 years it has also been formulated that in case of asthma suppression of inappropriate immune responses is either defective or overwhelmed. Several studies demonstrated that CD4+/CD25+ T cells are able to inhibit cytokine production and proliferation of TH2 cells in vitro and to suppress development of experimental asthma including AHR in mice [131, 132]; however, in asthmatic patients, the number of these cells appeared to be significantly decreased in peripheral blood, airway tissue, and BAL fluid. Furthermore, the percentage of CD4+CD25+ T cells in BAL correlated positively with FEV1 in asthmatic children [133–136]. The question why CD4+/CD25+ T cells are reduced in asthmatic patients has not been answered yet; however, it has been observed that these cells reveal a reduced response to the chemokines CCL1 and CXCL1 suggesting an impaired recruitment to the lung [137, 138].

Recent in vitro studies revealed a remarkable plasticity of Tregs that has so far not been interagted into the machnisms leading to allergic diseases such as asthma. When these cells are cultured with IL-6, they upregulate expression of the transcription factor ROR*γ*t and become IL-17 producing cells [139]. Furthermore, Tregs are able to induce CD4+CD25-Foxp3- T cells and can even induce their own transformation into IL-17 producing cells when TGF-*β* is absent and IL-6 is present [140]. Consequently, cd4+Foxp3+ T cells that produce IL-17 have been identified in humans and mice [141, 142]. Komatsu et al. even reported that after adoptive transfer of natural CD4+Foxp3+ T cells into lymphopenic or lymphoreplete recipients, a minor fraction enriched within the CD25(−) subset actually downregulated Foxp3 expression and started production of IL-17 and IFN-*γ* [143].

Interestingly, it has further been shown that Tregs also develop into T follicular helper (Tfh) cells that have initially been proposed as a separate T helper cell subpopulation failing to express cytokines and transcription factors, which are typical for TH1, TH2, or TH17 cells [144–147]. These recent studies demonstrate the enormous plasticity of T helper cells and which is attended by the challenge to classify different T helper cell lineages.

## 6. TH1 Cells in Asthma Pathology

TH1 cells, as cells that typically direct antibacterial or antifungal immune responses, mediate the activation of macrophages and clearance of intracellular pathogens by the production of IFN-*γ*, whereas cytokines secreted by TH2 cells are responsible for the production of IgE, the recruitment of eosinophils, and clearance of extracellular parasites [148, 149]. Thus, TH1 cells were believed to be responsible for organizing the immune response against bacteria, fungi, and viruses, while TH2 cells were mainly considered to play a role in defending against parasites. 

In the 1990s, the “hygiene hypothesis” came up, where allergic epidemics concluded that the western life style with its lack of TH1-triggering factors (e.g., exposure to microbial components) was responsible for the increase occurrence of allergic atopic diseases. Thus, the reduction of microbial burden reflected by the diminished production of IL-12 and interferon by natural immunity cells attenuate TH1 and enhance TH2 cell responses. Concerning the increased prevalence of atopic allergic diseases in Western countries, the predominance of TH2 responses to “innocuous” antigens became rational. Supportively, the occurrence of some infections in childhood reduces the risk for later atopy [150]. Moreover the prevalence of allergy is reduced in patients suffering from the TH1 driven multiple sclerosis [151]. Especially, the prevalence of asthma is on a rise in “westernized” countries with higher hygienic and socioeconomic standard [152]. Thus, manifold studies have shown that during the pathogenesis of asthma TH2 cells initiate inflammatory responses leading to AHR, the hallmark of asthma, [4, 153, 154]. Mice with deficient TH2 cytokine expression, induced by gene deletion or the usage of TH2 cytokine antagonists, showed diminished AHR and eosinophilic inflammation [9, 11]. The other subset of T helper cells, the TH1 cells, were however proposed to counteract TH2-mediated cell responses as seen in models of parasitic infection [155]. The described virtue of TH1 cells to counteract TH2 cell activities was subsequently reported for bronchial asthma. Enhancement of the TH1 response by local administration of IL-12 or IL-18 [156, 157] or the infection of TH1 promoting mycobacteria [147] or *Listeria* [158] diminished the development of allergic asthma, probably by causing impaired development of TH2 responsiveness. In a mouse model of experimental asthma, adoptive transfer of OVA specific TH1 cells reversed in a concentration-dependent manner TH2 cell derived IL-4 production, BAL eosinophilia and bronchial hyperresponsiveness [159, 160]. The underlying mechanism was hypothesized to be partly mediated by IFN-*γ*. IFN-*γ* is the ‘signature' cytokine of TH1 cells and its production is mediated by the tissue-specific transcription factor T-bet that is involved in the development of TH1 cells from native T cells [149]. Beside IFN-*γ*, TH1 cells produce LT-*α* and IL-2. These cytokines activate macrophages, natural killer cells, and CD8+ cytolytic cells and promote IgG2a class switching [155]. IFN-*γ* induces the expression of IL-12 by antigen presenting cells (APC) and thus promotes TH1 differentiation [155]. Actually, IFN-*γ* promotes the expression of the *β*-chain of IL-12 receptor through T-bet, indicating the importance of IFN-*γ* on IL-12-mediated effects on TH1 cells [161, 162]. Blocking of IFN-*γ* reversed the TH1 cell inhibition of TH2 cell-induced AHR [159]. Thus, based on these observations, promoting TH1 responses, in order to suppress allergic asthma, was proposed as an approach towards asthma therapy. However, while some studies could not show airway hyperresponsiveness after adoptive transfer of OVA specific TH1 cells [163–165], some other works demonstrated a distinct induction of AHR [166]. Consequently, the therapeutic utility of TH1 cells and their cytokines in allergic asthma became at least questionable. Moreover, TH1 responses together with TH2 responses may even act in combination to augment each other's activity to induce airway inflammation and AHR [164, 167]. Concordantly, in a mouse model of allergic asthma TH1 and TH2 cells were recruited together to the airways after sensitization with the model antigen OVA. However, TH1 cells were not only predominantly found early after the challenge; the presence of TH1 seems moreover to be a prerequisite for the recruitment of TH2 cells to the airways [168]. 

In the airways of asthmatic patients TH1 cells, increased levels of IFN-*γ*- and IFN-*γ*-dependent signaling molecules also have been detected beside TH2 cells and TH2 cytokines [169]. Hence, also patients suffering from allergic asthma displayed mixed T helper cell responses, reflected by the occurrence of TH2 specific IL-13 and of TH1 specific IFN-*γ* [170, 171]. Consequently, the TH1/TH2 paradigm appears to be more complex and the role of TH1 cells is much more controversial. Numerous studies were subsequently performed to evaluate the exact role of TH1 cells in allergic asthma and their interplay with TH2 cells.

The adoptive transfer of TH1 cells in a mouse model was capable to induce airway neutrophilia, accompanied with a dense perivascular and interstitial accumulation of small lymphocytes [166]. Concerning the function of TH1 cells in mucus hypersecretion, as one feature of bronchial asthma, observations were different. Since the adoptive transfer of antigen-specific TH1 cells induced mucus but much less than did TH2 cells [164], mucus hypersecretion was totally absent in a study by Cohn et al. [163]. In contrast to TH2 cells, TH1 cells produce high amounts of IFN-*γ* but low levels of IL-4, IL-5, and IL-13 [166]. Especially, IL-13 is involved in goblet cell metaplasia and thus in mucus hypersecretion [4]. By using recombinant cytokines Ford et al. [167] could demonstrate that IFN-*γ* possesses double-sided effects on IL-13-induced lung injury: on the one hand IFN-*γ* inhibited IL-13 dependent goblet cell hyperplasia and infiltration of eosinophils and neutrophils into the airways. On the other hand, combination of IL-13 and IFN-*γ* even enhanced peribronchial and alveolar inflammation as well as numbers of NK cells and IL-6 levels in BAL fluids. In addition, cells expressing high levels of molecules involved in antigen presentation such as MHC class II, CD11c, and CD68 were elevated [165]. However, TH1 cell-induced inflammation and induction of AHR appear to depend neither on IL-13 nor on IL-4 [166].

IL-18 was initially identified as a factor enhancing IFN-*γ* production from TH1 cells stimulated with anti-CD3 and IL-12 [172]. IL-18 is abundantly stored in the epithelial cells of various organs, including the lung [173]. Cellular IL-18 responsiveness is determined by the expression of the IL-18 receptor, a heterodimeric complex consisting of IL-18R*α* and IL-18R*β*. Upon IL-12 stimulation, native T cells develop into TH1 cells, expressing the IL-18R*α* [174]. Thus, endogenous interleukin-18 (IL-18) might play a critical role in the induction of bronchial asthma by TH1 cells through IL-18-dependent IFN-*γ* production [175, 176]. In this respect, Sugimoto and colleagues [176] demonstrated that the intranasal application of antigen and IL-18 induced AHR and airway inflammation, accompanied by the production of a mast cell similar cytokine profile, including GM-CSF, TNF-*α*, IL-9, IL-13, RANTES, and MIP-1*α* by reactivation of TH1 memory cells. In line with Cui et al. [166], neutralization of IL-13 did not reverse TH1 cell induced AHR. 

IFN-*γ* itself was initially regarded as an antiallergenic cytokine with the ability to counteract IL-4 as well as by reducing the proliferation of TH2 cells [177]. However, several studies could show that INF-*γ* activates eosinophils in vitro [178] or stimulates the recruitment of inflammatory cells by inducing ICAM-1 expression [179]. The role of IFN-*γ* is less clear. On the one hand administration of an anti-INF-*γ* Ab reversed the development of AHR and airway neutrophilia in mice receiving OVA specific TH1 cells [175, 180]. On the other hand, the study by Takaoka and colleagues [181] demonstrated that anti-IFN-*γ* Ab treatment did not reveal an inhibitory effect on TH1 mediated airway neutrophilia and AHR. 

Although TH1-dominated immune responses were originally considered to play a prominent role in autoimmune diseases, an impact of TH1 cells in rather TH2-dominated disorders, such as allergic bronchial asthma, is obvious. At least, the typical TH-1 cytokine IFN-*γ* has already been shown to play a role in the development of AHR and in aggravating inflammatory events in asthma. Nevertheless, further studies are required to fully elucidate the role of TH1 cells and especially of IFN-*γ* in asthma pathology. 

## 7. iNKT Cells in Asthma Pathology

Although not belonging to the group of T helper cells, a subgroup of natural killer T (NKT) cells has to be referred to in this paper, since by producing a number of the prementioned cytokines involved in asthma pathogenesis. A few years ago, invariant NKT (iNKT) cells have been described as a new subpopulation of T cells. Since these cells express not only the T cell receptor (TCR) but also the NK cell receptors NK1.1 or Ly49 in mice and CD161 in humans, current research defines these cells as a unique and evolutionally conserved group of T cells. These iNKT cells recognize glycolipid antigens through a TCR that is composed of an invariant TCR *α* chain and a TCR *β* chain with restricted variability [182–184]. In contrast to the typical cytokine profiles secreted by T helper cells these iNKT cells, which also may express CD4, are capable of producing large amounts of IL-4, IL-13, and IFN-*γ* after being stimulated via the TCR [185]. Due to these unique features, iNKT cells have soon been implicated in immunological disorders such as the development of allergic diseases. Thus, in iNKT cell-deficient *β*2 microglobulin knock-out mice, Yoshimoto et al. demonstrated that these cells are essential for the induction of IL-4-producing TH2 cells and consequently for the IL-4 dependent production of IgE, suggesting that iNKT cells are the source for the initial IL-4 required for TH2 cell development [186]. In contrast, mice lacking iNKT cells due to CD1- or J*α*18-deficiency developed the phenotype of experimental asthma after sensitization to and challenge with aerosolized OVA. However, in comparison to wild type animals IL-4 and IL-13 levels as well as eosinophil numbers in BAL fluid and IgE serum titers were significantly lower, whereas development of AHR was completely absent, indicating involvement of iNKT cells in the immunopathology of this disease. Most interestingly, the lack of AHR could be restored by adoptive transfer of IL-4/IL-13 producing iNKT cells [187, 188]. While these data demonstrated the importance of iNKT cells for the development of allergic disorders in mice, two studies further suggest contribution of these cells to human allergic asthma. On the one hand, Sen et al. figured out that the bronchial mucosa of asthmatic patients reveal massive accumulation of iNKT cells expressing the chemokine receptor CCR9 and CD226 in large amounts. Additionally, these cells are capable of inducing a strong TH2 bias upon CD226 engagement [189]. On the other hand, Akbari et al. reported that more than 60% of CD3+ or CD4+ T cells from patients suffering from moderate-to-severe asthma were IL-4/IL-13 producing iNKT cells expressing the V*α*24 TCR and CD4 [190]. The question how exactly iNKT cells fit into the large picture of asthma-immunopathology remains unanswered and is even complicated by the recent observations that iNKT cells additionally produce IL-9, IL-17, and IL-22 [191–193].

## 8. Conclusion

Taken together, with the identification of new T helper cell subsets, investigation of the mechanisms underlying asthma pathogenesis took another step forward. However, this insight also implicated a number of continuative questions. What exactly are the effects of TH9 or TH17 cells in asthma pathogenesis? Do TH22 or TH17/TH2 cells also participate in these processes? How do these cells communicate with each other and with infiltrated cells of the immune system or with structural cells of the airway wall? Do these new “non-TH2-type” T helper cells only play a role in special subtypes of asthma? Or are they even responsible for directing the disease process to a special development? The list of open questions that arised from the identification of new T helper cells in asthma could be continued and represents a task for future research in the field of asthma. But, it is also a chance to cut the Gordian knot of asthma phenotype diversity; these new T helper cell subpopulations could also represent new targets for therapeutic intervention in allergic diseases such as asthma.

## References

[1] Bousquet J, Jeffery PK, Busse WW, Johnson M, Vignola AM (2000). Asthma. From bronchoconstriction to airways inflammation and remodeling. *American Journal of Respiratory and Critical Care Medicine*.

[2] Wegmann M (2009). Th2 cells as targets for therapeutic intervention in allergic bronchial asthma. *Expert Review of Molecular Diagnostics*.

[3] Messi M, Giacchetto I, Nagata K, Lanzavecchia A, Natoli G, Sallusto F (2003). Memory and flexibility of cytokine gene expression as separable properties of human TH1 and TH2 lymphocytes. *Nature Immunology*.

[4] Wills-Karp M, Luyimbazi J, Xu X (1998). Interleukin-13: central mediator of allergic asthma. *Science*.

[5] Bacharier LB, Geha RS (2000). Molecular mechanisms of IgE regulation. *Journal of Allergy and Clinical Immunology*.

[6] Renauld J-C (2001). New insights into the role of cytokines in asthma. *Journal of Clinical Pathology*.

[7] Rosenberg HF, Phipps S, Foster PS (2007). Eosinophil trafficking in allergy and asthma. *Journal of Allergy and Clinical Immunology*.

[8] Wills-Karp M (2004). Interleukin-13 in asthma pathogenesis. *Immunological Reviews*.

[9] Brusselle GG, Kips JC, Tavernier JH (1994). Attenuation of allergic airway inflammation in IL-4 deficient mice. *Clinical and Experimental Allergy*.

[10] Walter DM, McIntire JJ, Berry G (2001). Critical role for IL-13 in the development of allergen-induced airway hyperreactivity. *Journal of Immunology*.

[11] Foster PS, Hogan SP, Ramsay AJ, Matthaei KI, Young IG (1996). Interleukin 5 deficiency abolishes eosinophilia, airways hyperreactivity, and lung damage in a mouse asthma model. *Journal of Experimental Medicine*.

[12] Wegmann M, Hauber HP (2010). Experimental approaches towards allergic asthma therapy-murine asthma models. *Recent Patents on Inflammation and Allergy Drug Discovery*.

[13] Borish LC, Nelson HS, Lanz MJ (1999). Interleukin-4 receptor in moderate atopic asthma. A phase I/II randomized, placebo-controlled trial. *American Journal of Respiratory and Critical Care Medicine*.

[14] Leckie MJ, Ten Brinke A, Khan J (2000). Effects of an interleukin-5 blocking monoclonal antibody on eosinophils, airway hyper-responsiveness, and the late asthmatic response. *Lancet*.

[15] Wenzel S, Wilbraham D, Fuller R, Getz EB, Longphre M (2007). Effect of an interleukin-4 variant on late phase asthmatic response to allergen challenge in asthmatic patients: results of two phase 2a studies. *Lancet*.

[16] Sel S, Wegmann M, Sel S (2007). Immunomodulatory effects of viral TLR ligands on experimental asthma depend on the additive effects of IL-12 and IL-10. *Journal of Immunology*.

[17] Monteyne P, Renauld J-C, Van Broeck J, Dunne DW, Brombacher F, Coutelier J-P (1997). IL-4-independent regulation of in vivo IL-9 expression. *Journal of Immunology*.

[18] Shimbara A, Christodoulopoulos P, Soussi-Gounni A (2000). IL-9 and its receptor in allergic and nonallergic lung disease: increased expression in asthma. *Journal of Allergy and Clinical Immunology*.

[19] Erpenbeck VJ, Hohlfeld JM, Volkmann B (2003). Segmental allergen challenge in patients with atopic asthma leads to increased IL-9 expression in bronchoalveolar lavage fluid lymphocytes. *Journal of Allergy and Clinical Immunology*.

[20] Veldhoen M, Uyttenhove C, van Snick J (2008). Transforming growth factor-*β* ’reprograms’ the differentiation of T helper 2 cells and promotes an interleukin 9-producing subset. *Nature Immunology*.

[21] Dardalhon V, Awasthi A, Kwon H (2008). IL-4 inhibits TGF-*β*-induced Foxp3^+^ T cells and, together with TGF-*β*, generates IL-9^+^ IL-10^+^ Foxp3^−^ effector T cells. *Nature Immunology*.

[22] Zhu J, Min B, Hu-Li J (2004). Conditional deletion of Gata3 shows its essential function in T_H_1-T_H_2 responses. *Nature Immunology*.

[23] Houssiau FA, Schandene L, Stevens M (1995). A cascade of cytokines is responsible for IL-9 expression in human T cells: involvement of IL-2, IL-4, and IL-10. *Journal of Immunology*.

[24] Jäger A, Dardalhon V, Sobel RA, Bettelli E, Kuchroo VK (2009). Th1, Th17, and Th9 effector cells induce experimental autoimmune encephalomyelitis with different pathological phenotypes. *Journal of Immunology*.

[25] Hoffmann KF, Cheever AW, Wynn TA (2000). IL-10 and the dangers of immune polarization: excessive type 1 and type 2 cytokine responses induce distinct forms of lethal immunopathology in murine schistosomiasis. *Journal of Immunology*.

[26] Li C, Corraliza I, Langhorne J (1999). A defect in interleukin-10 leads to enhanced malarial disease in Plasmodium chabaudi chabaudi infection in mice. *Infection and Immunity*.

[27] Gazzinelli RT, Oswald IP, James SL, Sher A (1992). IL-10 inhibits parasite killing and nitrogen oxide production by IFN-*γ*- activated macrophages. *Journal of Immunology*.

[28] Van Leeuwen BH, Martinson ME, Webb GC, Young IG (1989). Molecular organization of the cytokine gene cluster, involving the human IL-3, IL-4, IL-5, and GM-CSF genes, on human chromosome 5. *Blood*.

[29] Postma DS, Bleecker ER, Amelung PJ (1995). Genetic susceptibility to asthma—bronchial hyperresponsiveness coinherited with a major gene for atopy. *New England Journal of Medicine*.

[30] Renaud J-C, Kermouni A, Vink A, Louahed J, Van Snick J (1995). Interleukin-9 and its receptor: involvement in mast cell differentiation and T cell oncogenesis. *Journal of Leukocyte Biology*.

[31] Gordon JR, Burd PR, Galli SJ (1990). Mast cells as a source of multifunctional cytokines. *Immunology Today*.

[32] Bradding P (1996). Human mast cell cytokines. *Clinical and Experimental Allergy*.

[33] Hültner L, Kölsch S, Stassen M (2000). In activated mast cells, IL-1 up-regulates the production of several Th2-related cytokines including IL-9. *Journal of Immunology*.

[34] Gounni AS, Nutku E, Koussih L (2000). IL-9 expression by human eosinophils: regulation by IL-1*β* and TNF-*α*. *Journal of Allergy and Clinical Immunology*.

[35] Hultner L, Druez C, Moeller J (1990). Mast cell growth-enhancing activity (MEA) is structurally related and functionally identical to the novel mouse T cell growth factor P40/TCGFIII (interleukin 9). *European Journal of Immunology*.

[36] Louahed J, Kermouni A, Van Snick J, Renauld J-C (1995). IL-9 induces expression of granzymes and high-affinity IgE receptor in murine T helper clones. *Journal of Immunology*.

[37] Van Snick J, Goethals A, Renauld J-C (1989). Cloning and characterization of a cDNA for a new mouse T cell growth factor [P40]. *Journal of Experimental Medicine*.

[38] Gessner A, Blum H, Rollinghoff M (1993). Differential regulation of IL-9-expression after infection with Leishmania major in susceptible and resistant mice. *Immunobiology*.

[39] Temann U-A, Geba GP, Rankin JA, Flavell RA (1998). Expression of interleukin 9 in the lungs of transgenic mice causes airway inflammation, mast cell hyperplasia, and bronchial hyperresponsiveness. *Journal of Experimental Medicine*.

[40] McLane MP, Haczku A, Van De Rijn M (1998). Interleukin-9 promotes allergen-induced eosinophilic inflammation and airway hyperresponsiveness in transgenic mice. *American Journal of Respiratory Cell and Molecular Biology*.

[41] Vink A, Warnier G, Brombacher F, Renauld J-C (1999). Interleukin 9-induced in vivo expansion of the B-1 lymphocyte population. *Journal of Experimental Medicine*.

[42] Louahed J, Zhou Y, Lee Maloy W (2001). Interleukin 9 promotes influx and local maturation of eosinophils. *Blood*.

[43] Gounni AS, Gregory B, Nutku E (2000). Interleukin-9 enhances interleukin-5 receptor expression, differentiation, and survival of human eosinophils. *Blood*.

[44] Dong Q, Louahed J, Vink A (1999). IL-9 induces chemokine expression in lung epithelial cells and baseline airway eosinophilia in transgenic mice. *European Journal of Immunology*.

[45] Little FF, Cruikshank WW, Center DM (2001). IL-9 stimulates release of chemotactic factors from human bronchial epithelial cells. *American Journal of Respiratory Cell and Molecular Biology*.

[46] Louahed J, Toda M, Jen J (2000). Interleukin-9 upregulates mucus expression in the airways. *American Journal of Respiratory Cell and Molecular Biology*.

[47] Longphre M, Li D, Gallup M (1999). Allergen-induced IL-9 directly stimulates mucin transcription in respiratory epithelial cells. *Journal of Clinical Investigation*.

[48] Nicolaides NC, Holroyd KJ, Ewart SL (1997). Interleukin 9: a candidate gene for asthma. *Proceedings of the National Academy of Sciences of the United States of America*.

[49] Abdelilah SG, Latifa K, Esra N (2001). Functional expression of IL-9 receptor by human neutrophils from asthmatic donors: role in IL-8 release. *Journal of Immunology*.

[50] Rankin JA, Picarella DE, Geba GP (1996). Phenotypic and physiologic characterization of transgenic mice expressing interleukin 4 in the lung: lymphocytic and eosinophilic inflammation without airway hyperreactivity. *Proceedings of the National Academy of Sciences of the United States of America*.

[51] Levitt RC, McLane MR, MacDonald D (1999). IL-9 pathway in asthma: new therapeutic targets for allergic inflammatory disorders. *Journal of Allergy and Clinical Immunology*.

[52] Bergeron C, Al-Ramli W, Hamid Q (2009). Remodeling in asthma. *Proceedings of the American Thoracic Society*.

[53] Doherty T, Broide D (2007). Cytokines and growth factors in airway remodeling in asthma. *Current Opinion in Immunology*.

[54] Reader JR, Hyde DM, Schelegle ES (2003). Interleukin-9 induces mucous cell metaplasia independent of inflammation. *American Journal of Respiratory Cell and Molecular Biology*.

[55] Vermeer PD, Harson R, Einwalter LA, Moninger T, Zabner J (2003). Interleukin-9 induces goblet cell hyperplasia during repair of human airway epithelia. *American Journal of Respiratory Cell and Molecular Biology*.

[56] Whittaker L, Niu N, Temann U-A (2002). Interleukin-13 mediates a fundamental pathway for airway epithelial mucus induced by CD4 T cells and interleukin-9. *American Journal of Respiratory Cell and Molecular Biology*.

[57] Steenwinckel V, Louahed J, Orabona C (2007). IL-13 mediates in vivo IL-9 activities on lung epithelial cells but not on hematopoietic cells. *Journal of Immunology*.

[58] Van Den Brûle S, Heymans J, Havaux X (2007). Profibrotic effect of IL-9 overexpression in a model of airway remodeling. *American Journal of Respiratory Cell and Molecular Biology*.

[59] Doherty TA, Soroosh P, Broide DH, Croft M (2009). CD4+ cells are required for chronic eosinophilic lung inflammation but not airway remodeling. *American Journal of Physiology*.

[60] Boxall CB, Holgate ST, Davies DE (2006). The contribution of transforming growth factor-*β* and epidermal growth factor signalling to airway remodelling in chronic asthma. *European Respiratory Journal*.

[61] Murphy CA, Langrish CL, Chen Y (2003). Divergent Pro- and antiinflammatory roles for IL-23 and IL-12 in joint autoimmune inflammation. *Journal of Experimental Medicine*.

[62] Cua DJ, Sherlock J, Chen Y (2003). Interleukin-23 rather than interleukin-12 is the critical cytokine for autoimmune inflammation of the brain. *Nature*.

[63] Langrish CL, Chen Y, Blumenschein WM (2005). IL-23 drives a pathogenic T cell population that induces autoimmune inflammation. *Journal of Experimental Medicine*.

[64] Steinman L (2007). A brief history of T_H_17
, the first major revision in the T_H_1-T_H_2
hypothesis of T cell-mediated tissue damage. *Nature Medicine*.

[65] Kreymborg K, Etzensperger R, Dumoutier L (2007). IL-22 is expressed by Th17 cells in an IL-23-dependent fashion, but not required for the development of autoimmune encephalomyelitis. *Journal of Immunology*.

[66] Wong CK, Ho CY, Ko FWS (2001). Proinflammatory cytokines (IL-17, IL-6, IL-18 and IL-12) and Th cytokines (IFN-*γ*, IL-4, IL-10 and IL-13) in patients with allergic asthma. *Clinical and Experimental Immunology*.

[67] Molet S, Hamid Q, Davoine F (2001). IL-17 is increased in asthmatic airways and induces human bronchial fibroblasts to produce cytokines. *Journal of Allergy and Clinical Immunology*.

[68] Pène J, Chevalier S, Preisser L (2008). Chronically inflamed human tissues are infiltrated by highly differentiated Th17 lymphocytes. *Journal of Immunology*.

[69] Bullens DMA, Truyen E, Coteur L (2006). IL-17 mRNA in sputum of asthmatic patients: linking T cell driven inflammation and granulocytic influx?. *Respiratory Research*.

[70] Laan M, Cui Z-H, Hoshino H (1999). Neutrophil recruitment by human IL-17 via C-X-C chemokine release in the airways. *Journal of Immunology*.

[71] Laan M, Lötvall J, Chung KF, Lindén A (2001). IL-17-induced cytokine release in human bronchial epithelial cells in vitro: role of mitogen-activated protein (MAP) kinases. *British Journal of Pharmacology*.

[72] Jones CE, Chan K (2002). Interleukin-17 stimulates the expression of interleukin-8, growth-related oncogene-*α*, and granulocyte-colony-stimulating factor by human airway epithelial cells. *American Journal of Respiratory Cell and Molecular Biology*.

[73] Kawaguchi M, Kokubu F, Kuga H (2001). Modulation of bronchial epithelial cells by IL-17. *Journal of Allergy and Clinical Immunology*.

[74] Kawaguchi M, Kokubu F, Odaka M (2004). Induction of granulocyte-macrophage colony-stimulating factor by a new cytokine, ML-1 (IL-17F), via Raf I-MEK-ERK pathway. *Journal of Allergy and Clinical Immunology*.

[75] Henness S, Johnson CK, Ge Q, Armour CL, Hughes JM, Ammit AJ (2004). IL-17A augments TNF-*α*-induced IL-6 expression in airway smooth muscle by enhancing mRNA stability. *Journal of Allergy and Clinical Immunology*.

[76] Hoshino H, Laan M, Sjöstrand M, Lötvall J, Skoogh B-E, Lindén A (2000). Increased elastase and myeloperoxidase activity associated with neutrophil recruitment by IL-17 in airways in vivo. *Journal of Allergy and Clinical Immunology*.

[77] Traves SL, Donnelly LE (2008). Th17 cells in airway diseases. *Current Molecular Medicine*.

[78] Gavett SH, Chen X, Finkelman F, Wills-Karp M (1994). Depletion of murine CD4+ T lymphocytes prevents antigen-induced airway hyperreactivity and pulmonary eosinophilia. *American Journal of Respiratory Cell and Molecular Biology*.

[79] Renzi PM, Yang JP, Diamantstein T, Martin JG (1996). Effects of depletion of cells bearing the interleukin-2 receptor on immunoglobulin production and allergic airway responses in the rat. *American Journal of Respiratory and Critical Care Medicine*.

[80] Prause O, Bozinovski S, Anderson GP, Lindén A (2004). Increased matrix metalloproteinase-9 concentration and activity after stimulation with interleukin-17 in mouse airways. *Thorax*.

[81] McKinley L, Alcorn JF, Peterson A (2008). TH17 cells mediate steroid-resistant airway inflammation and airway hyperresponsiveness in mice. *Journal of Immunology*.

[82] Hellings PW, Kasran A, Liu Z (2003). Interleukin-17 orchestrates the granulocyte influx into airways after allergen inhalation in a mouse model of allergic asthma. *American Journal of Respiratory Cell and Molecular Biology*.

[83] Oda N, Canelos PB, Essayan DM, Plunkett BA, Myers AC, Huang S-K (2005). Interleukin-17F induces pulmonary neutrophilia and amplifies antigen-induced allergic response. *American Journal of Respiratory and Critical Care Medicine*.

[84] Chakir J, Shannon J, Molet S (2003). Airway remodeling-associated mediators in moderate to severe asthma: effect of steroids on TGF-*β*, IL-11, IL-17, and type I and type III collagen expression. *Journal of Allergy and Clinical Immunology*.

[85] Wong CK, Lun SWM, Ko FWS (2009). Activation of peripheral Th17 lymphocytes in patients with asthma. *Immunological Investigations*.

[86] Zhao Y, Yang J, Gao Y-D, Guo W (2009). Th17 immunity in patients with allergic asthma. *International Archives of Allergy and Immunology*.

[87] Cosmi L, Maggi L, Santarlasci V (2010). Identification of a novel subset of human circulating memory CD4^+^ T cells that produce both IL-17A and IL-4. *Journal of Allergy and Clinical Immunology*.

[88] Weaver CT, Hatton RD, Mangan PR, Harrington LE (2007). IL-17 family cytokines and the expanding diversity of effector T cell lineages. *Annual Review of Immunology*.

[89] Song C, Luo L, Lei Z (2008). IL-17-producing alveolar macrophages mediate allergic lung inflammation related to asthma. *Journal of Immunology*.

[90] Happel KI, Zheng M, Young E (2003). Cutting edge: roles of toll-like receptor 4 and IL-23 in IL-17 expression in response to Klebsiella pneumoniae infection. *Journal of Immunology*.

[91] Ferretti S, Bonneau O, Dubois GR, Jones CE, Trifilieff A (2003). Il-17, produced by lymphocytes and neutrophils, is necessary for lipopolysaccharide-induced airway neutrophilia: IL-15 as a possible trigger. *Journal of Immunology*.

[92] Stark MA, Huo Y, Burcin TL, Morris MA, Olson TS, Ley K (2005). Phagocytosis of apoptotic neutrophils regulates granulopoiesis via IL-23 and IL-17. *Immunity*.

[93] Pichavant M, Goya S, Meyer EH (2008). Ozone exposure in a mouse model induces airway hyperreactivity that requires the presence of natural killer T cells and IL-17. *Journal of Experimental Medicine*.

[94] Doganci A, Sauer K, Karwot R, Finotto S (2005). Pathological role of IL-6 in the experimental allergic bronchial asthma in mice. *Clinical Reviews in Allergy and Immunology*.

[95] Mukhopadhyay S, Hoidal JR, Mukherjee TK (2006). Role of TNF*α* in pulmonary pathophysiology. *Respiratory Research*.

[96] Brightling C, Berry M, Amrani Y (2008). Targeting TNF-*α*: a novel therapeutic approach for asthma. *Journal of Allergy and Clinical Immunology*.

[97] Hymowitz SG, Filvaroff EH, Yin J (2001). IL-17s adopt a cystine knot fold: structure and activity of a novel cytokine, IL-17F, and implications for receptor binding. *EMBO Journal*.

[98] Starnes T, Robertson MJ, Sledge G (2001). Cutting edge: IL-17F, a novel cytokine selectively expressed in activated T cells and monocytes, regulates angiogenesis and endothelial cell cytokine production. *Journal of Immunology*.

[99] Kawaguchi M, Kokubu F, Matsukura S (2003). Induction of C-X-C chemokines, growth-related oncogene *α* expression, and epithelial cell-derived neutrophil-activating protein-78 by ML-1 (interleukin-17F) involves activation of raf1-mitogen-activated protein kinase kinase-extracellular signal-regulated kinase 1/2 pathway. *Journal of Pharmacology and Experimental Therapeutics*.

[100] Kawaguchi M, Onuchic LF, Li X-D (2001). Identification of a novel cytokine, ML-1, and its expression in subjects with asthma. *Journal of Immunology*.

[101] Wurster AL, Rodgers VL, Satoskar AR (2002). Interleukin 21 is a T helper (Th) cell 2 cytokine that specifically inhibits the differentiation of naive Th cells into interferon *γ*-producing Th1 cells. *Journal of Experimental Medicine*.

[102] Vinuesa CG, Tangye SG, Moser B, Mackay CR (2005). Follicular B helper T cells in antibody responses and autoimmunity. *Nature Reviews Immunology*.

[103] Korn T, Bettelli E, Gao W (2007). IL-21 initiates an alternative pathway to induce proinflammatory T_H_17 cells. *Nature*.

[104] Nurieva R, Yang XO, Martinez G (2007). Essential autocrine regulation by IL-21 in the generation of inflammatory T cells. *Nature*.

[105] Suto A, Kashiwakuma D, Kagami S-I (2008). Development and characterization of IL-21-producing CD4+ T cells. *Journal of Experimental Medicine*.

[106] Zhou L, Ivanov II, Spolski R (2007). IL-6 programs TH-17 cell differentiation by promoting sequential engagement of the IL-21 and IL-23 pathways. *Nature Immunology*.

[107] Avery DT, Ma CS, Bryant VL (2008). STAT3 is required for IL-21 induced secretion of IgE from human naive B cells. *Blood*.

[108] Wolk K, Kunz S, Asadullah K, Sabat R (2002). Cutting edge: immune cells as sources and targets of the IL-10 family members?. *Journal of Immunology*.

[109] Wolk K, Kunz S, Witte E, Friedrich M, Asadullah K, Sabat R (2004). IL-22 increases the innate immunity of tissues. *Immunity*.

[110] Aujla SJ, Chan YR, Zheng M (2008). IL-22 mediates mucosal host defense against Gram-negative bacterial pneumonia. *Nature Medicine*.

[111] Zheng Y, Valdez PA, Danilenko DM (2008). Interleukin-22 mediates early host defense against attaching and effacing bacterial pathogens. *Nature Medicine*.

[112] Duhen T, Geiger R, Jarrossay D, Lanzavecchia A, Sallusto F (2009). Production of interleukin 22 but not interleukin 17 by a subset of human skin-homing memory T cells. *Nature Immunology*.

[113] Knappe A, Hör S, Wittmann S, Fickenscher H (2000). Induction of a novel cellular homolog of interleukin-10, AK155, by transformation of T lymphocytes with herpesvirus saimiri. *Journal of Virology*.

[114] Wakashin H, Hirose K, Maezawa Y (2008). IL-23 and Th17 cells enhance Th2-cell-mediated eosinophilic airway inflammation in mice. *American Journal of Respiratory and Critical Care Medicine*.

[115] Cheung PFY, Wong CK, Lam CWK (2008). Molecular mechanisms of cytokine and chemokine release from eosinophils activated by IL-17A, IL-17F, and IL-23: implication for Th17 lymphocytes-mediated allergic inflammation. *Journal of Immunology*.

[116] Tato CM, Laurence A, O’Shea JJ (2006). Helper T cell differentiation enters a new era: le roi est mort; vive le roi!. *Journal of Experimental Medicine*.

[117] Pan G, French D, Mao W (2001). Forced expression of murine IL-17E induces growth retardation, jaundice, a Th2-biased response, and multiorgan inflammation in mice. *Journal of Immunology*.

[118] Kim MR, Manoukian R, Yeh R (2002). Transgenic overexpression of human IL-17E results in eosinophilia, B-lymphocyte hyperplasia, and altered antibody production. *Blood*.

[119] Hurst SD, Muchamuel T, Gorman DM (2002). New IL-17 family members promote Th1 or Th2 responses in the lung: in vivo function of the novel cytokine IL-25. *Journal of Immunology*.

[120] Owyang AM, Zaph C, Wilson EH (2006). Interleukin 25 regulates type 2 cytokine-dependent immunity and limits chronic inflammation in the gastrointestinal tract. *Journal of Experimental Medicine*.

[121] Fallon PG, Ballantyne SJ, Mangan NE (2006). Identification of an interleukin (IL)-25-dependent cell population that provides IL-4, IL-5, and IL-13 at the onset of helminth expulsion. *Journal of Experimental Medicine*.

[122] Ballantyne SJ, Barlow JL, Jolin HE (2007). Blocking IL-25 prevents airway hyperresponsiveness in allergic asthma. *Journal of Allergy and Clinical Immunology*.

[123] Tamachi T, Maezawa Y, Ikeda K (2006). IL-25 enhances allergic airway inflammation by amplifying a TH2 cell-dependent pathway in mice. *Journal of Allergy and Clinical Immunology*.

[124] Stock P, Lombardi V, Kohlrautz V, Akbari O (2009). Induction of airway hyperreactivity by IL-25 is dependent on a subset of invariant NKT cells expressing IL-17RB. *Journal of Immunology*.

[125] Angkasekwinai P, Chang SH, Thapa M, Watarai H, Dong C (2010). Regulation of IL-9 expression by IL-25 signaling. *Nature Immunology*.

[126] Smith DE (2010). IL-33: a tissue derived cytokine pathway involved in allergic inflammation and asthma. *Clinical and Experimental Allergy*.

[127] Liew FY, Pitman NI, McInnes IB (2010). Disease-associated functions of IL-33: the new kid in the IL-1 family. *Nature Reviews in Immunology*.

[128] Sakaguchi S, Ono M, Setoguchi R (2006). Foxp3+CD25+CD4+ natural regulatory T cells in dominant self-tolerance and autoimmune disease. *Immunological Reviews*.

[129] Akdis CA, Akdis M (2009). Mechanisms and treatment of allergic disease in the big picture of regulatory T cells. *Journal of Allergy and Clinical Immunology*.

[130] Robinson DS (2009). Regulatory T cells and asthma. *Clinical and Experimental Allergy*.

[131] Ling EM, Smith T, Nguyen XD (2004). Relation of CD4+CD25+ regulatory T-cell suppression of allergen-driven T-cell activation to atopic status and expression of allergic disease. *Lancet*.

[132] Presser K, Schwinge D, Wegmann M (2008). Coexpression of TGF-beta1 and IL-10 enables regulatory T cells to completely suppress airway hyperreactivity. *Journal of Immunology*.

[133] Grindebacke H, Wing K, Andersson AC, Suri-Payer E, Rak S, Rudin A (2004). Defective suppression of Th2 cytokines by CD4CD25 regulatory T cells in birch allergics during birch pollen season. *Clinical Experimental Allergy*.

[134] Akdis M, Verhagen J, Taylor A (2004). Immune responses in healthy and allergic individuals are characterized by a fine balance between allergen-specific T regulatory 1 and T helper 2 cells. *Journal of Experimental Medicine*.

[135] Hartl D, Koller B, Mehlhorn AT (2007). Quantitative and functional impairment of pulmonary CD4+CD25hi regulatory T cells inpediatric asthma. *Journal of Allergy and Clinical Immunology*.

[136] Lin Y-L, Shieh C-C, Wang J-Y (2008). The functional insufficiency of human CD4^+^CD25^high^ T-regulatory cells in allergic asthma is subjected to TNF-*α* modulation. *Allergy*.

[137] Nguyen KD, Fohner A, Booker JD, Dong C, Krensky AM, Nadeau KC (2008). XCL1 enhances regulatory activities of CD4^+^CD25^high^CD127^low/-^ T cells in human allergic asthma. *Journal of Immunology*.

[138] Nguyen KD, Vanichsarn C, Fohner A, Nadeau KC (2009). Selective deregulation in chemokine signaling pathways of CD4^+^CD25^hi^CD127^lo/-^ regulatory T cells in human allergic asthma. *Journal of Allergy and Clinical Immunology*.

[139] Yang XO, Nurieva R, Martinez GJ (2008). Molecular antagonism and plasticity of regulatory and inflammatory T cell programs. *Immunity*.

[140] Xu L, Kitani A, Fuss I, Strober W (2007). Cutting edge: regulatory T cells induce CD4+CD25 -Foxp3- T cells or are self-induced to become Th17 cells in the absence of exogenous TGF-*β*. *Journal of Immunology*.

[141] Lochner M, Peduto L, Cherrier M (2008). In vivo equilibrium of proinflammatory IL-17+ and regulatory IL-10+ Foxp3+ RORgamma t+ T cells. *Journal of Experimental Medicine*.

[142] Voo KS, Wang Y-H, Santori FR (2009). Identification of IL-17-producing FOXP3+ regulatory T cells in humans. *Proceedings of the National Academy of Sciences of the United States of America*.

[143] Komatsu N, Mariotti-Ferrandiz ME, Wang Y, Malissen B, Waldmann H, Hori S (2009). Heterogeneity of natural Foxp3+ T cells: a committed regulatory T-cell lineage and an uncommitted minor population retaining plasticity. *Proceedings of the National Academy of Sciences of the United States of America*.

[144] King C, Tangye SG, Mackay CR (2008). T follicular helper (TFH) cells in normal and dysregulated immune responses. *Annual Review of Immunology*.

[145] Vogelzang A, McGuire HM, Yu D, Sprent J, Mackay CR, King C (2008). A fundamental role for interleukin-21 in the generation of T follicular helper cells. *Immunity*.

[146] Nurieva RI, Chung Y, Hwang D (2008). Generation of T follicular helper cells is mediated by interleukin-21 but independent of T helper 1, 2, or 17 cell lineages. *Immunity*.

[147] Tsuji M, Komatsu N, Kawamoto S (2009). Preferential generation of follicular B helper T cells from Foxp3+ T cells in gut Peyer’s patches. *Science*.

[148] Ansel KM, Djuretic I, Tanasa B, Rao A (2006). Regulation of Th2 differentiation and Il4 locus accessibility. *Annual Review of Immunology*.

[149] Szabo SJ, Sullivan BM, Peng SL, Glimcher LH (2003). Molecular mechanisms regulating Th1 immune responses. *Annual Review of Immunology*.

[150] Shirakawa T, Enomoto T, Shimazu S-I, Hopkin JM (1997). The inverse association between tuberculin responses and atopic disorder. *Science*.

[151] Tang L, Benjaponpitak S, DeKruyff RH, Umetsu DT (1998). Reduced prevalence of allergic disease in patients with multiple sclerosis is associated with enhanced IL-12 production. *Journal of Allergy and Clinical Immunology*.

[152] Székely JI, Pataki A (2009). Recent findings on the pathogenesis of bronchial asthma—part II. The role of hormonal predisposition, environmental influences and conditioning leading to bronchial asthma. *Acta Physiologica Hungarica*.

[153] Kuperman DA, Huang X, Koth LL (2002). Direct effects of interleukin-13 on epithelial cells cause airway hyperreactivity and mucus overproduction in asthma. *Nature Medicine*.

[154] Nakanishi A, Morita S, Iwashita H (2001). Role of gob-5 in mucus overproduction and airway hyperresponsiveness in asthma. *Proceedings of the National Academy of Sciences of the United States of America*.

[155] Abbas AK, Murphy KM, Sher A (1996). Functional diversity of helper T lymphocytes. *Nature*.

[156] Gavett SH, O’Hearn DJ, Li X, Huang S-K, Finkelman FD, Wills-Karp M (1995). Interleukin 12 inhibits antigen-induced airway hyperresponsiveness, inflammation, and Th2 cytokine expression in mice. *Journal of Experimental Medicine*.

[157] Hofstra CL, Van Ark I, Hofman G, Kool M, Nijkamp FP, Van Oosterhout AJM (1998). Prevention of Th2-like cell responses by coadministration of IL-12 and IL-18 is associated with inhibition of antigen-induced airway hyperresponsiveness, eosinophilia, and serum IgE levels. *Journal of Immunology*.

[158] Yeung VP, Gieni RS, Umetsu DT, DeKruyff RH (1998). Heat-killed Listeria monocytogenes as an adjuvant converts established murine Th2-dominated immune responses into Th1-dominated responses. *Journal of Immunology*.

[159] Huang T-J, MacAry PA, Eynott P (2001). Allergen-specific Th1 cells counteract efferent Th2 cell-dependent bronchial hyperresponsiveness and eosinophilic inflammation partly via IFN-*γ*. *Journal of Immunology*.

[160] Bodey KJ, Semper AE, Redington AE (1999). Cytokine profiles of BAL T cells and T-cell clones obtained from human asthmatic airways after local allergen challenge. *Allergy*.

[161] Mullen AC, High FA, Hutchins AS (2001). Role of T-bet in commitment of TH1 cells before IL- 12-dependent selection. *Science*.

[162] Afkarian M, Sedy JR, Yang J (2002). T-bet is a STAT1-induced regulator of IL-12R expression in naïve CD4+ Tcells. *Nature Immunology*.

[163] Cohn L, Tepper JS, Bottomly K (1998). Cutting edge: IL-4-independent induction of airway hyperresponsiveness by Th2, but not Th1, cells. *Journal of Immunology*.

[164] Hansen G, Berry G, DeKruyff RH, Umetsu DT (1999). Allergen-specific Th1 cells fail to counterbalance Th2 cell-induced airway hyperreactivity but cause severe airway inflammation. *Journal of Clinical Investigation*.

[165] Sawicka E, Zuany-Amorim C, Manlius C (2003). Inhibition of Th1- and Th2-mediated airway inflammation by the sphingosine 1-phosphate receptor agonist FTY720. *Journal of Immunology*.

[166] Cui J, Pazdziorko S, Miyashiro JS (2005). TH1-mediated airway hyperresponsiveness independent of neutrophilic inflammation. *Journal of Allergy and Clinical Immunology*.

[167] Ford JG, Rennick D, Donaldson DD (2001). IL-13 and IFN-*γ*: interactions in lung inflammation. *Journal of Immunology*.

[168] Randolph DA, Carruthers CJL, Szabo SJ, Murphy KM, Chaplin DD (1999). Modulation of airway inflammation by passive transfer of allergen-specific Th1 and Th2 cells in a mouse model of asthma. *Journal of Immunology*.

[169] Krug N, Madden J, Redington AE (1996). T-cell cytokine profile evaluated at the single cell level in BAL and blood in allergic asthma. *American Journal of Respiratory Cell and Molecular Biology*.

[170] Holtzman MJ, Sampath D, Castro M, Look DC, Jayaraman S (1996). The one-two of T helper cells: does interferon-*γ* knock out the Th2 hypothesis for asthma?. *American Journal of Respiratory Cell and Molecular Biology*.

[171] Hol BE, Bruinier B, Lutter R, Jansen HM, Out TA (1996). Cytokine production by T-cell clones from bronchoalveolar lavage fluid of patients with asthma and healthy subjects. *European Respiratory Journal*.

[172] Okamura H, Tsutsui H, Komatsu T (1995). Cloning of a new cytokine that induces IFN-*γ* production by T cells. *Nature*.

[173] Cameron LA, Taha RA, Tsicopoulos A (1999). Airway epithelium expresses interleukin-18. *European Respiratory Journal*.

[174] Yoshimoto T, Takeda K, Tanaka T (1998). IL-12 up-regulates IL-18 receptor expression on T cells, Th1 cells, and B cells: synergism with IL-18 for IFN-*γ* production. *Journal of Immunology*.

[175] Hayashi N, Yoshimoto T, Izuhara K, Matsui K, Tanaka T, Nakanishi K (2007). T helper 1 cells stimulated with ovalbumin and IL-18 induce airway hyperresponsiveness and lung fibrosis by IFN-*γ* and IL-13 production. *Proceedings of the National Academy of Sciences of the United States of America*.

[176] Sugimoto T, Ishikawa Y, Yoshimoto T, Hayashi N, Fujimoto J, Nakanishi K (2004). Interleukin 18 acts on memory T helper cells type 1 to induce airway inflammation and hyperresponsiveness in a naive host mouse. *Journal of Experimental Medicine*.

[177] Maggi E, Parronchi P, Manetti R (1992). Reciprocal regulatory effects of IFN-*γ* and IL-4 on the in vitro development of human Th1 and Th2 clones. *Journal of Immunology*.

[178] Valerius T, Repp R, Kalden JR, Platzer E (1990). Effects of IFN on human eosinophils in comparison with other cytokines. A novel class of eosinophil activators with delayed onset of action. *Journal of Immunology*.

[179] Look DC, Rapp SR, Keller BT, Holtzman MJ (1992). Selective induction of intercellular adhesion molecule-1 by interferon-*γ* in human airway epithelial cells. *American Journal of Physiology*.

[180] Kumar RK, Webb DC, Herbert C, Foster PS (2006). Interferon-*γ* as a possible target in chronic asthma. *Inflammation and Allergy*.

[181] Takaoka A, Tanaka Y, Tsuji T (2001). A critical role for mouse CXC chemokine(s) in pulmonary neutrophilia during Th type 1-dependent airway inflammation. *Journal of Immunology*.

[182] Bendelac A, Rivera MN, Park S-H, Roark JH (1997). Mouse CD1-specific NK1 T cells: development, specificity, and function. *Annual Review of Immunology*.

[183] Taniguchi M, Harada M, Kojo S, Nakayama T, Wakao H (2003). The regulatory role of Valpha14 NKT cells in innate and acquired immune response. *Annual Reviews of Immunology*.

[184] Kronenberg M (2005). Toward an understanding of NKT cell biology: progress and paradoxes. *Annual Review of Immunology*.

[185] Godfrey DI, MacDonald HR, Kronenberg M, Smyth MJ, Van Kaer L (2004). NKT cells: what’s in a name?. *Nature Reviews Immunology*.

[186] Yoshimoto T, Bendelac A, Watson C, Hu-Li J, Paul WE (1995). Role of NK1.1+ T cells in a TH2 response and in immunoglobulin E production. *Science*.

[187] Smiley ST, Kaplan MH, Grusby MJ (1997). Immunoglobulin E production in the absence of interleukin-4-secreting CD1-dependent cells. *Science*.

[188] Akbari O, Stock P, Meyer E (2003). Essential role of NKT cells producing IL-4 and IL-13 in the development of allergen-induced airway hyperreactivity. *Nature Medicine*.

[189] Sen Y, Yongyi B, Yuling H (2005). V alpha 24-invariant NKT cells from patients with allergic asthma express CCR9 at high frequency and induce Th2 bias of CD3+ T cells upon CD226 engagement. *Journal of Immunology*.

[190] Akbari O, Faul JL, Hoyte EG (2006). CD4+ invariant T-cell-receptor+ natural killer T cells in bronchial asthma. *New England Journal of Medicine*.

[191] Coquet JM, Chakravarti S, Kyparissoudis K (2008). Diverse cytokine production by NKT cell subsets and identification of an IL-17-producing CD4^−^NK1. 1^−^ NKT cell population. *Proceedings of the National Academy of Sciences of the United States of America*.

[192] Rachitskaya AV, Hansen AM, Horai R (2008). Cutting edge: NKT cells constitutively express IL-23 receptor and RORgammat and rapidly produce IL-17 upon receptor ligation in an IL-6-independent fashion. *Journal of Immunology*.

[193] Goto M, Murakawa M, Kadoshima-Yamaoka K (2009). Murine NKT cells produce Th17 cytokine interleukin-22. *Cellular Immunology*.

